# Research of the Pre-Processing Strategy Influence on the Tribological Properties of PEI Processed by Fused Filament Fabrication Technology

**DOI:** 10.3390/ma16134527

**Published:** 2023-06-22

**Authors:** Gerhard Mitaľ, Ivan Gajdoš, Emil Spišák

**Affiliations:** Department of Technology, Materials and Computer Supported Production, Faculty of Mechanical Engineering, Technical University of Košice, Mäsiarska 74, 04001 Košice, Slovakia; gerhard.mital@tuke.sk (G.M.); emil.spisak@tuke.sk (E.S.)

**Keywords:** polyetherimide, fused filament fabrication, 3D printing, path generation strategy, abrasive wear, tribological properties

## Abstract

The aim of this study was to investigate the effect of Fused Filament Fabrication (FFF) layer generation strategies on abrasive wear resistance and compare the material loss of PEI (polyetherimide) plastic specimens based on different specimen building strategies. The study also compares a newly proposed path generation strategy (parallel paths in layers with 0.25 mm displacement of alternate layers) with samples from a previous study where samples were printed without displacement of alternate layers, i.e., layers stacked perpendicularly to each other. The primary focus was on the weight loss due to abrasive wear before and after the test. The tests were conducted on a tribometer constructed according to ASTM G65/16 standards using dry sand. Two printing directions were examined: X (longitudinal) and Z (portrait) orientations. For X construction, three orientations of deposition path generation were utilized, resulting in three samples for each orientation (nine samples in total for X construction). The same approach was applied to Z construction, resulting in another nine samples. In total, 18 samples were produced and tested. The deposited infill path width was 0.5 mm, and the layer thickness used in printing was 0.254 mm. Garnet abrasive Fe_3_Al_2_(SiO_4_)_3_ was employed in this test. Analysis of the experimental data revealed a relationship between the construction method (X and Z orientations) and the variation in different orientations (1X–3X and 1Z–3Z). The research results can be categorized as overall and partial. The overall results indicate poorer wear resistance for 1X–3X and 1Z–3Z specimens, while the partial results illustrate the findings within each individual specimen.

## 1. Introduction

FFF as one of the additive manufacturing technologies has undergone a great boom in the last decades and has found applications ranging from hobby applications to highly demanding industrial applications. Compared to conventional technologies, FFF (Fused Filament Fabrication) offers the possibility of producing parts by adding material instead of removing it, but it also has its limitations, especially when it comes to small batch production or small part sizes [1,2]. While many studies have been devoted to the actual printing process and mechanical properties of printed parts, due to the limitations resulting from the nature of FFF technology [3,4,5], the tribological properties of printed models have only been studied marginally and on a limited range of materials [6]. The most widely and frequently utilized materials for FFF were also the subject of numerous studies, investigating their tribological properties. Among such materials we can include acrylonitrile butadiene styrene (ABS) [7,8,9,10,11,12], polyactic acid (PLA) [13,14,15], polycarbonate/acrylonitrile butadiene styrene (PC-ABS) [16], polyamide (PA) [17], polyether ether ketone (PEEK) [18], and polyethersulfone (PES) [19].

A study by Jadwiga Pisula et al. [7] investigated the contact wear of spur gears made by the FFF method from ABS, polyetherimide (PEI), and PEEK, finding that the greatest wear was observed for gears made from PEI material. The hardness of the material decreased due to the operating temperature of the loaded gear. Studies by Sencer Sureyya Karabeyoglu et al. [9], Investigated the dependence of texture and filling density on wear and wear rate, friction coefficient, wear mechanism, and microscopic wear characteristics of parts printed from ABS. The results revealed that a grid pattern of high infill density and a honeycomb pattern of low infill density showed the lowest wear rate and lowest coefficient of friction compared to the rectilinear pattern. Infill pattern and density affected the wear rate behavior of specimens directly.

Wenzheng Wu et al. [19], Investigated fiber-reinforced thermoplastic polymer composites based on PES and basalt fibers. By improving the interfacial bonding properties between the fiber and resin matrix with a silane coupling agent, the mass wear and the wear scar width decreased by 80% and 45%, respectively, compared with those for pure PES. Some studies indicated the nature of plastic part behavior during abrasive wear such as S. Perepelkina et al. [14], Where analysis of experimental data showed that changing the 3D printing (filling) settings during part construction made of PLA had a significant effect not only on the strength and stiffness of the parts but also on the surface quality, which influences the tribological properties of the tribological couples. The results of these investigations make it possible to select the optimal 3D printing settings depending on the desired tribological properties of the parts, such as friction and wear coefficients, the ability of the thermoplastic material to soften by heating, and to form under pressure.

Singh R. et al. [17], Stated that the use of functional prototypes based on fused deposition modeling (FDM) for wear resistance applications is currently limited mainly due to selective availability of filament material. Several thermoplastic-based fillers have been reinforced by various research groups to improve mechanical properties. Current research with three particle sizes added suggests that the pin reinforced with double particle size shows better wear properties, followed by triple particle size and ABS reinforcement with the first particle type.

In the experiment of Lin L. et al. [18], A PEEK coating material reinforced with carbon fibers (CF) was prepared on a pure PEEK substrate. The results show that the friction coefficient and wear rate are strongly dependent on the direction of wear action with respect to the orientation of the print paths. The values are much lower when the wear direction was perpendicular to the carbon fibers. Comparative analyses of the worn surfaces show that an effective distribution of shear stress on the carbon fibers can contribute to a large reduction in friction and wear.

Tribological properties of PEI were the object of research in studies by Gu, Y. et al. [20] and Bijwe et al. In these cases, PEI was processed and utilized by other technologies than 3D printing. Observed wear performance of PEI was significantly affected by operating parameters such as load, speed, temperature, and sliding duration and depends largely on the type of wear. Neat PEI polymer performed best in abrasive and erosive wear modes [21].

The application of PEI as a high-performance polymer material in FFF additive manufacturing is gaining ground and research into the properties and performance of the parts made of PEI by FFF is becoming crucial. Much of the research is focused on mechanical properties [5,22,23]. On the other hand, tribological properties of 3D printed parts made of PEI are investigated only marginally.

A study by Mitaľ et al. [24] investigated the influence of processing parameters on wear performance of 3D printed parts. Abrasion resistance of PEI material showed the suitability of the chosen build orientation and deposition strategy for the production of parts by additive manufacturing, depending on the required tribological properties such as friction coefficient and wear behavior at 3D printing technological parameters such as layer height of 0.254 mm and filament width of 0.5 mm. The results of the study show that the wear continuity and frictional force depend on the trajectory traversed in the model orientation of the fabrication. The magnitude of wear (material loss) ranged from 0.451% to 0.809%. It was shown that the weight loss of the specimen under load was on average greater for the chosen fiber orientation strategy in the Z direction than in the X direction [20].

The purpose of this research study is to enhance the knowledge about abrasion resistance of PEI 3D parts, especially the influence of the path generation strategy. To fill this gap, in the present work, the effect of the sample building orientation and in-layer deposition strategy has been studied. This work is envisaged to help the designer in considering both the manufacturing settings through FFF along with the real abrasion resistance of parts made of PEI by FFF.

## 2. Materials and Methods

### 2.1. Measurements and Equipment

**Figure 1:**
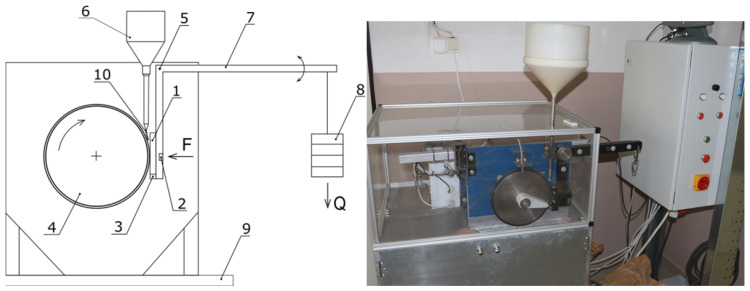
Principle of friction and wear measurement (G65/16)—**left**; tribometer—**right**.

The testing apparatus (Figure 1) contains a tribological pair consisting of a rotating disc and a specimen mounted in a holder.

In a tribometer with a rotating rubber disc mounted on a base (9), abrasive wear conditions are created by particles (10) which are discharged from a reservoir (6) between the sample (1) and the rotating disc (4).

During testing, the wear rate of the surface layers is monitored as a function of the sliding path and load. At the same time, the effect of different abrasive particles on the wear of the specimens can be investigated. It is possible to change the load magnitude and the rotation speed of the wheel (so-called sliding speed). The load magnitude is generated by the load unit (5, 7, 8). The device allows samples with different physical and mechanical properties to be tested. During testing, the time history of the specimen temperature, normal force, friction force, and sliding distance can be continuously recorded. The device can be set to automatically terminate the test after a certain number of rotations. The test process can be controlled and monitored via computer.

**Figure 2:**
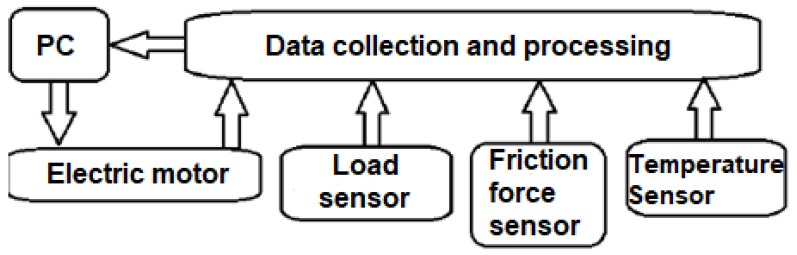
Scheme of data processing during the test.

Prior to the testing process, it is possible to adjust the magnitude of the normal force by fixing and positioning the weight on the lever, whereby the normal force is measured using the force sensor (2) which is located in the arm (7). The arm holding the test specimen is pivotally mounted by means of a pin (5). To measure the frictional force between the specimen (1) and the rotating disc (4), a frictional force sensor (3) is located in the load (8). The control of the sliding speed (rotation) is realized based on the desired value of the time course of the movement or the number of cycles. The advantage of this technical solution is that it has the ability to record the friction force, the normal force, and the temperature of the sample and to convert this data into a tabular form. Figure 2 shows a schematic of the flow and extent of data acquisition and processing.

### 2.2. Experimental Parameters

Experimental measurements were carried out according to ASTM G65/16 (Standard Test Method for Measuring Abrasion Using the Dry Sand/Rubber Wheel Apparatus) at an ambient temperature of 21 °C and relative humidity of 64%.

To determine the wear rate as a function of specimen build direction and orientation of the deposition paths, 9 tests were performed for specimens with X orientation and 9 tests for specimens with Z orientation, using garnet Fe_3_Al_2_ (SiO_4_)_3_ abrasive. The same rubber disc was used for all tests to eliminate any differences in the chemical composition or hardness of the rubber. As a basis for determining wear, the weight loss of the samples was determined by weighing the samples on a precision balance with an accuracy of 0.0001 g before and after the test. The abrasive test results are reported as weight loss in grams for a particular test procedure according to the following formula [25]:

(1) $$\;mass\;loss=\frac{weight\;loss}{density\left(\frac{g}{cm^{3}}\right)}\times 1000$$

The advantage of this technical solution is that it allows the recording of friction and normal force and temperature of the sample during the test. The machine parameter settings during the test are shown in Table 1.

**Figure 3:**
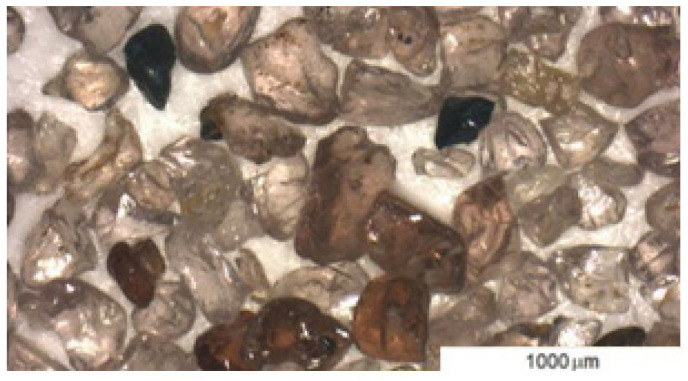
Garnet abrasive.

The three-point abrasive process involves loose particles that are free to move (abrasive). Garnet abrasive was used in this experiment. Garnet sand is a natural material [24]. The composition of the abrasive used is shown in Table 2. Figure 3 shows a snapshot of the abrasive used.

### 2.3. Pre-Processing and Production of Experimental Samples

The most common material used in the production of models using FDM technology is ABS thermoplastic, PC polycarbonate and derivatives, polyphenyl sulfone PPSU/PPSF, and ULTEM 9085™ material [26].

**Figure 4:**
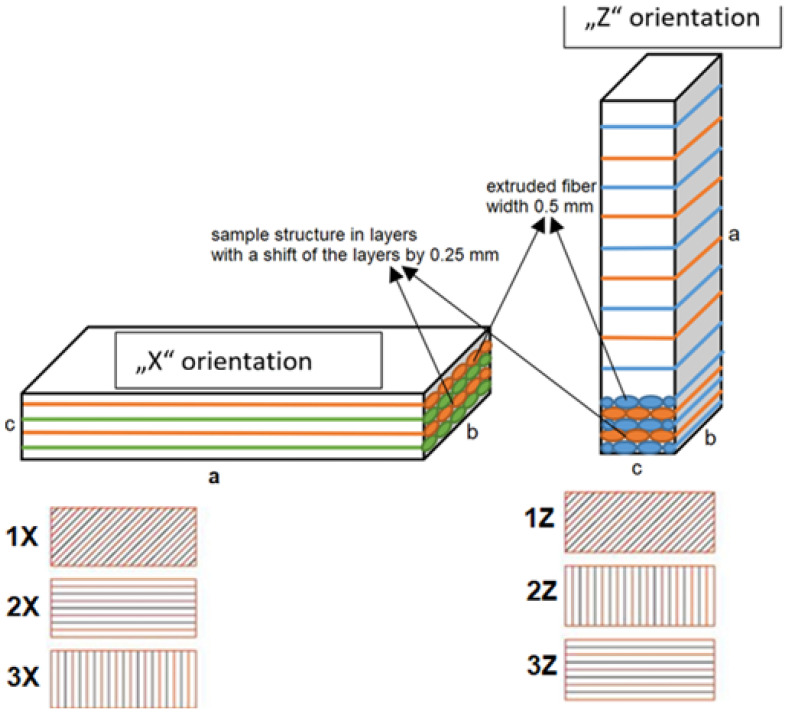
Layer deposition strategy in X and Z orientation (**top**) and orientation of deposited fibers in alternate layers (**bottom**).

The samples were made from PEI polymer (ULTEM 9085™) on a Fortus 400mc™ 3D printer at 380 °C nozzle temperature according to the scheme shown in Figure 4. All crucial parameters (nozzle temperature, chamber temperature, and layer height) were applied according to default process setting values valid for ULTEM9085™ in Stratassys Insight 10.1™ slicing software. Printing conditions and parameters are summarized in Table 3.

PEI is a high-temperature-resistant amorphous plastic with an amber-transparent coloration. The material meets the highest requirements for heat resistance (permanent operating temperatures up to 170 °C are possible depending on the application and type), and at the same time, thanks to its chemical composition, slows down combustion without the addition of further flame retardants, with very low smoke generation. It has a very high strength, which can be increased by adding glass or carbon fibers. The wide range of material properties is complemented by above-average dimensional stability and a low tendency to creep. All these properties make the material an ideal candidate for the automotive and aerospace industries. Thermoplastic is also popularly used in the telecommunications and medical technology sectors.

The specimens were in the shape of a 70 × 20 × 6 mm block (a × b × c), and the contact surface in contact with the rotating disc was the wall of the specimen with a dimension of 70 × 20 mm (Figure 4 top). The strategies used in the sample construction are shown in Figure 4, where the alternating layers (XY plane) when printing the sample were at the same angle but with a shift of the printing nozzle by 0.25 mm which provided a better filling of the sample volume and a denser structure [24]. The same construction procedure was used for both orientations.

Figure 4 (lower half) depicts the method used for printing samples in alternating layers. In this experiment, the alternating layers were printed at the same angle during sample formation, but the axis of the deposition path was shifted by 0.25 mm in a direction perpendicular to the deposition direction. In Figure 4 (top half), the odd layers are denoted in blue and green to enhance clarity in the schematic representation. Samples 1X, 2X, and 3X were deposited with the structure’s orientation labeled as X, while samples 1Z, 2Z, and 3Z were deposited with the structure’s orientation indicated as Z.

In the abrasive wear resistance test, three types of track fit orientation were applied to the specimen structure in the X and Z directions to investigate the weight loss of the material. Table 4 shows the specimen designations and the angles at which the specimen was formed.

## 3. Results and Discussion

**Figure 5:**
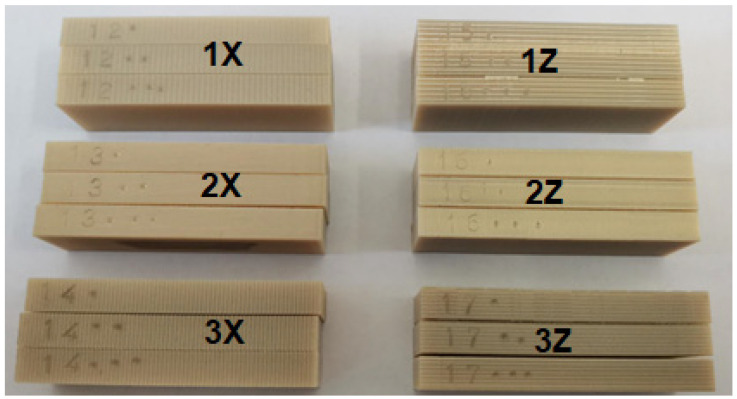
Samples used in the experiment.

The experimental results were evaluated as the value of weight loss and friction force waveform as a function of the traveled path for different orientations of specimen construction by FFF process. Two orientations of specimen construction (X and Z orientation) were made for this experiment. For each orientation, three identical methods of path deposition and layer formation were used, up to the desired specimen size. Three samples were made from each method, thus a total of 18 samples were tested (Figure 5).

### 3.1. Comparison of 1X–3X Samples with the Same Path Orientation without Displacement in the Intermediate Layers of Samples 2–4

**Figure 6:**
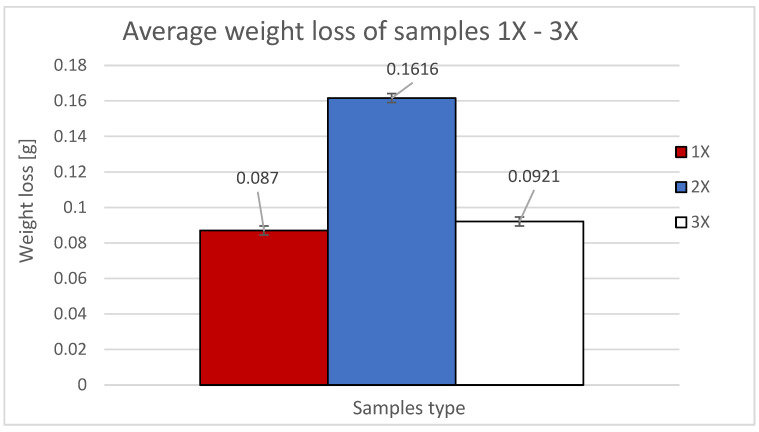
Weight losses and standard deviation of 1X, 2X and 3X samples.

The experimental results are presented as the weight loss of the sample as a function of the deposition orientation. Figure 6 shows the average weight loss of the samples used with X orientation.

**Figure 7:**
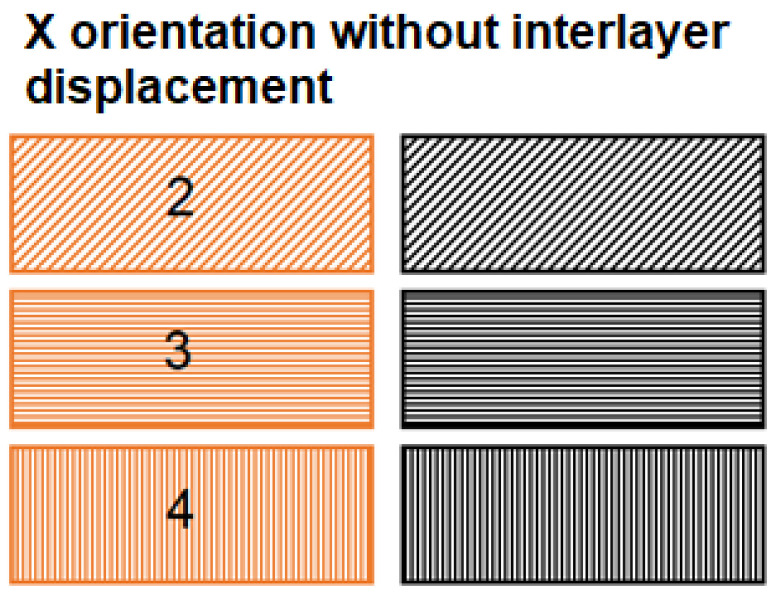
Path formation strategy in even (black) and odd (orange) structural layers of investigated samples with X orientation of deposited fibers [24].

Figure 7 shows the orientation of the path deposition in odd and even layers in samples from study [24]. These orientations are compared with newly produced samples with a denser structure. The denser structure was achieved by shifting the internalized layers by 0.25 mm, which ensured a higher weight of the printed samples in the X orientation and thus a denser interlayered sample structure.

**Figure 8:**
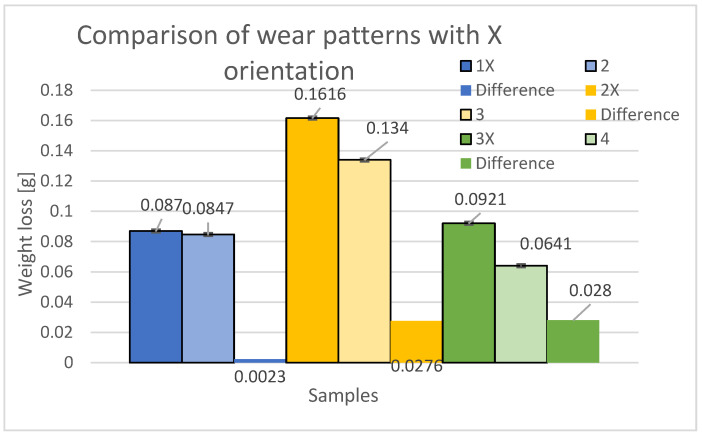
Comparison and difference and standard deviation of weight loss of 1X, 2X, and 3X samples with samples with the same orientation but without displacement in alternate layers of samples 2, 3, and 4 [24].

Figure 8 displays a comparison of mass differences between specimens in the X orientation and the material loss due to abrasive wear. The blue color represents the loss of samples 1X (new samples) and 2 (from the previous study). Within the 45° orientation, the specimen with a perpendicular build system in the alternate layers (printing paths directly above each other) proved to be more durable, exhibiting a 2.64% decrease in wear compared to specimen 1X, with a loss difference of 0.0023 g. The yellow color illustrates the losses of specimens 2X (new specimens) and 3 (from the previous study). Within the 0° orientation, the specimen with a perpendicular printing method in alternate layers (printing paths directly above each other) demonstrated higher durability, with a 17.08% decrease in wear compared to the 2X specimen, resulting in a loss difference of 0.0276 g. The green color showcases the losses of the 3X specimens (new specimens) and the 4 specimens (from the previous study). Within the 90° orientation, the specimen with perpendicular printing in alternate layers (printing paths directly above each other) was found to be more resistant, exhibiting a 30.4% decrease in wear compared to the 3X specimen, resulting in a loss difference of 0.0286 g. Based on this comparison, it was observed that there was a decrease in abrasive wear resistance for the specimens with a 0.25 mm offset in the alternate layers when printed in the X orientation. Thus, specimens with the alternating layers printed perpendicularly on top of each other (without interlayer offset) demonstrated higher resistance in all print path orientations.

**Figure 9:**
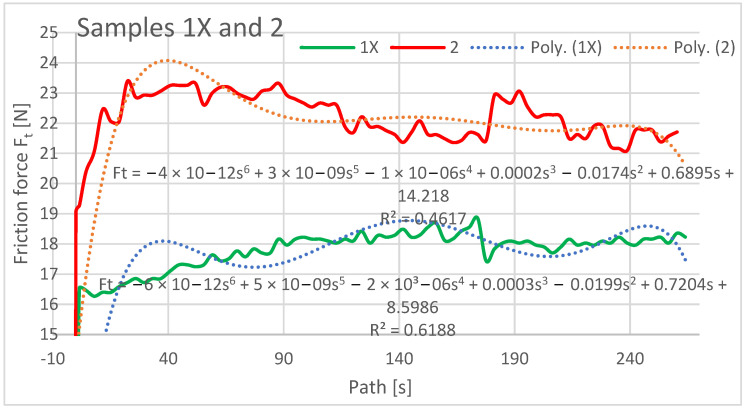
Plot of coefficient of friction versus path for samples 1X and 2.

**Figure 10:**
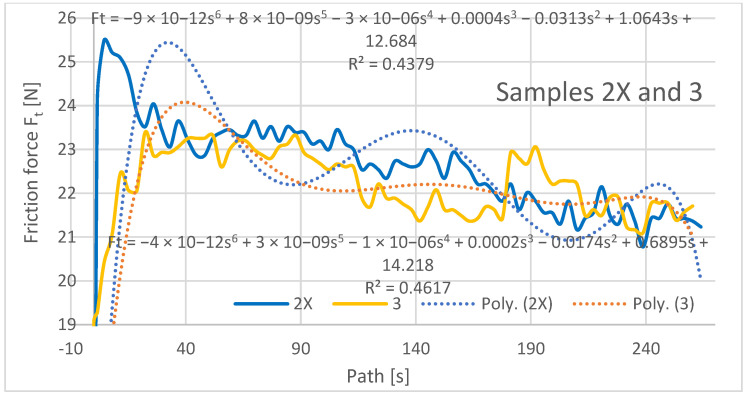
Plot of coefficient of friction versus path for samples 2X and 3.

**Figure 11:**
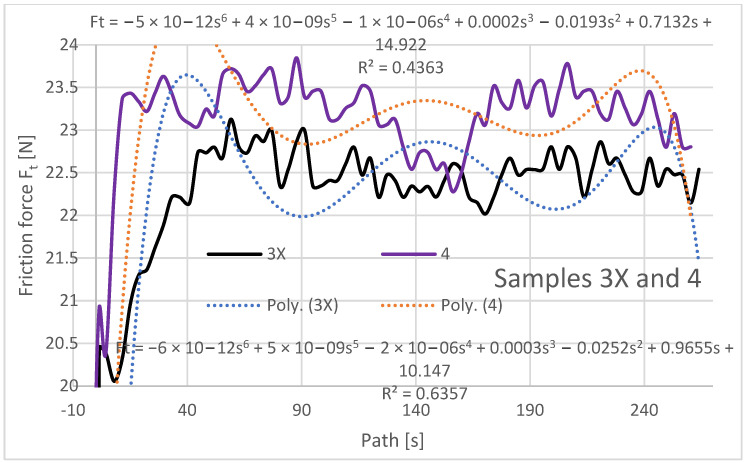
Plot of coefficient of friction versus path for samples 3X and 4.

The friction force was also evaluated as a dependent variable in the evaluation of the experimental data as a function of the path of the rubberized disc. The frictional force was read directly from the tribometer output data, and its evolution depends on several factors such as the mechanical properties of the material, contact pressure and friction, chemical interactions of the materials in the three contact tribosystem (disc, abrasive particles, specimen), and the contact time length. The resulting friction force curves are shown in Figure 9, Figure 10 and Figure 11 and are processed into separate graphical dependencies for better clarity.

The graphical dependencies of the samples with 1X–3X orientation show that they are less resistant to wear compared to samples 2–4. Samples 1X and 2 in the test showed similar material loss characteristics. This means that if the layers are applied at an angle of 45° from the direction of the abrasion flow that this does not have much effect on the wear, whether the deposition paths are overlapping or shifted with a 0.25 mm rasterization offset.

The highest frictional force was at the beginning of the abrasion test, which was due to the material putting up the most resistance to the frictional force at the beginning. It can be seen from the dependencies that the coefficient of friction from the initial value at the beginning of the experiment increases and stabilizes after a path of 40–50 m, where it then oscillates around a certain value until the end of the experiment. The lowest coefficient of friction at the beginning of the experiment can be observed for the Type 1X samples, which is also the lowest at the end along with the coefficient of friction for the Type 3 and 3X samples.

Within the graphical characterization of the friction forces, regression mathematical models of the given dependencies were also worked out, which indicate that there is negligible mathematical dependence between the compared variables, i.e., that it is mathematically meaningless to investigate the compared variables and to create mathematical models.

### 3.2. Comparison of 1Z–3Z Samples with the Same Fiber Orientation without Displacement in the Intermediate Layers of Samples 7, 8, and 10

**Figure 12:**
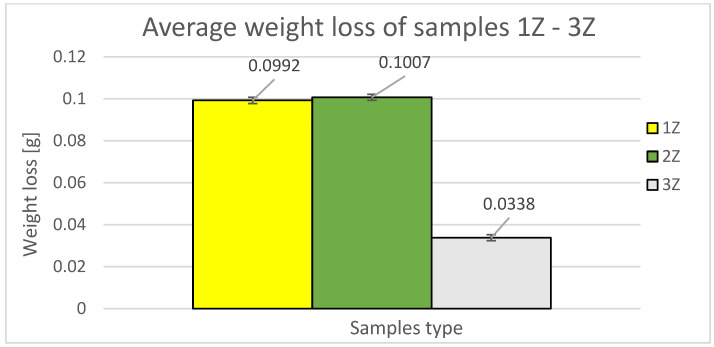
Weight losses and standard deviation of 1Z, 2Z, and 3Z samples.

The experimental results are presented as the weight loss of the sample as a function of the deposition orientation. Figure 12 shows the average weight loss of the samples used with Z orientation.

**Figure 13:**
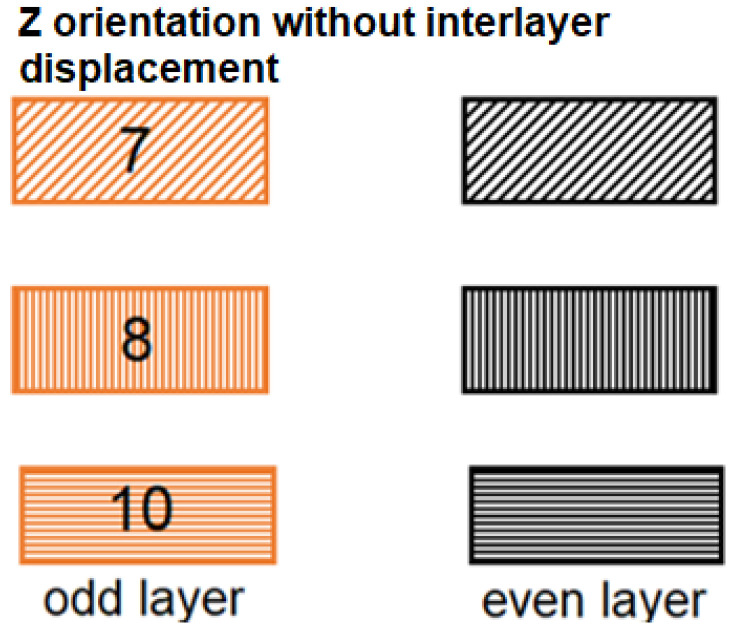
Path formation strategy in even (black) and odd (orange) structural layers of investigated samples with Z orientation of deposited fibers [24].

Figure 13 shows the orientation of the path deposition in odd and even layers in samples from study [24]. These orientations are also compared with new samples with a denser structure, where the denser structure was achieved by shifting the internalized layers by 0.25 mm, which provided a lower weight of the printed samples in the Z orientation and thus a more densely bonded sample structure.

**Figure 14:**
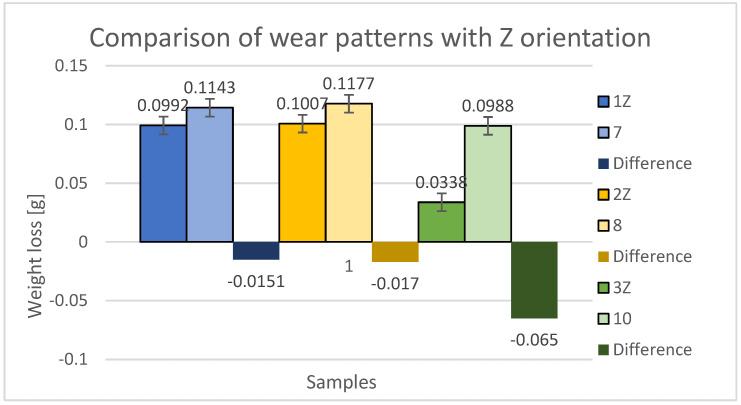
Comparison and difference of weight losses and standard deviation of samples 1Z, 2Z, and 3Z with samples with the same orientation but without displacement in the intermediate layers of samples 7, 8, and 10.

The graphical representation in Figure 14 provides a comparison of weight differences between specimens in the Z orientation and the material loss due to abrasive wear. The blue color indicates the loss of samples 1Z (new samples) and 7 (from the previous study). Within the 45° orientation, the specimen with perpendicular printing in alternating layers (printing paths directly above each other) demonstrated lower durability, with a 15.22% increase in wear compared to specimen 1Z and a loss difference of 0.0151 g.

The yellow color represents the losses of specimens 2Z (new specimens) and 8 (from the previous study). Within the 90° orientation, the specimen with a perpendicular construction system in alternate layers (fibers directly and with each other) also exhibited lower durability, with a 15.88% increase in wear compared to specimen 2Z, resulting in a loss difference of 0.017 g.

The green color shows the losses of specimens 3Z (new specimens) and 10 (from the previous study). Within the 0° orientation, the specimen with a perpendicular printing method in alternating layers (printing paths directly above each other) demonstrated even lower durability, with a 192.31% increase in wear compared to specimen 3Z, and a loss difference of 0.065 g.

Based on this aforementioned comparison, it was found that specimens built in the Z orientation, with a 0.25 mm offset of the alternating layers of fibers, exhibited higher abrasive wear resistance compared to specimens with a perpendicularly superimposed fiber deposition construction system. Among the trio of 1Z, 2Z, and 3Z, specimen 3Z demonstrated the lowest weight loss. For specimens 1Z and 2Z, weight loss was approximately the same. This indicates that the specimens produced in the Z orientation experienced a lower weight loss and thus a lower wear.

**Figure 15:**
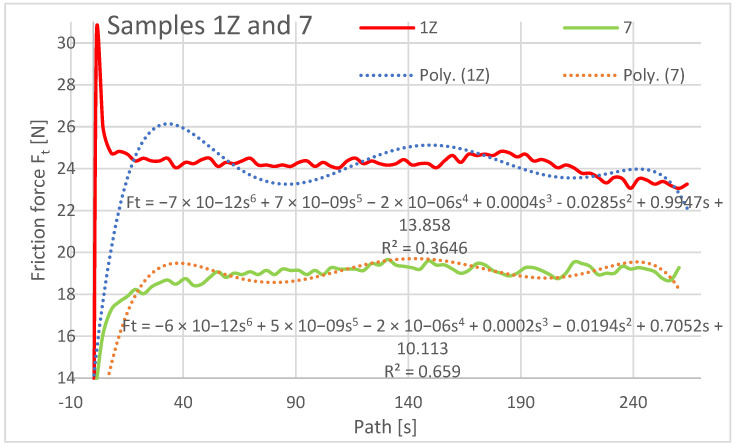
Plot of coefficient of friction versus path, samples 1Z and 7.

**Figure 16:**
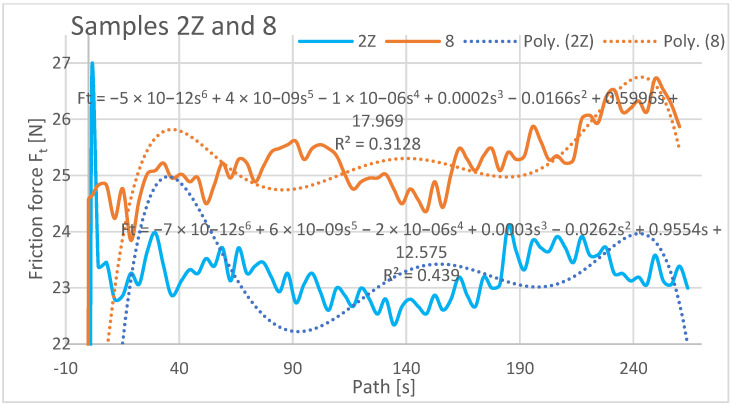
Plot of coefficient of friction versus path, samples 2Z and 8.

**Figure 17:**
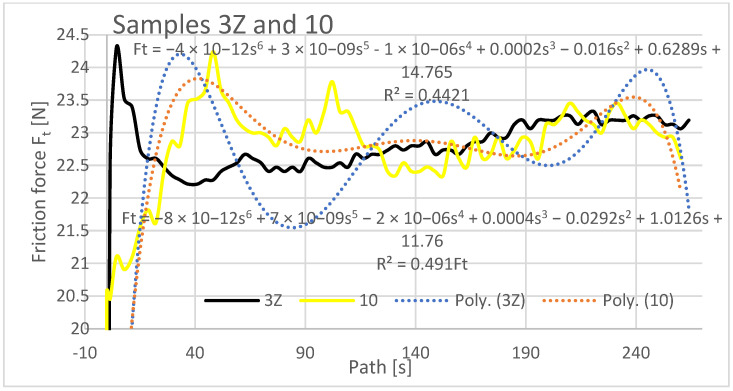
Graph of coefficient of friction versus path, samples 3Z and 10.

In the evaluation of the experimental data, the friction force was also evaluated as a dependent variable in the Z orientation depending on the traveled path of the rubberized disc. The friction force was read directly from the tribometer output data. The resulting friction force curves are shown separately in Figure 15, Figure 16 and Figure 17.

The graphical analysis of specimens with orientations 1Z to 3Z reveals their higher resistance to wear compared to specimens 7, 8, and 10. As weight loss is directly proportional to frictional force, specimens 3Z and 10 exhibited similar weight loss characteristics in the frictional force test. This suggests that depositing the layers at 0° does not significantly impact friction and wear. Similarly, a comparable friction and wear pattern occurs when the layers are deposited on top of each other with a 0.25 mm rasterization offset. Regarding samples 1Z, 7, 2Z, and 8, different friction forces were observed. The highest friction was recorded for sample 8 (ranging from 24 N to 26.5 N) and 1Z (ranging from 24.8 N to 23.3 N). Specimens with orientations 2Z and 7 exhibited lower frictional forces, while the lowest friction was observed for specimen 7 (ranging from 18 N to 19.4 N) from the previous study. In the current study, the specimen with 3Z orientation demonstrated the lowest friction.

It is important to note the history of frictional forces in the compared specimens of the X and Z series in the current study. The frictional force did not change significantly when comparing specimens with the same orientations. Initially, the highest friction force was observed at the beginning of the abrasion test, indicating that the material offered the greatest resistance to the applied force. The friction coefficient increased from the initial value at the start of the experiment and stabilized after a distance of 10–20 m. It then oscillated around a certain value until the end of the experiment, with a slight increase towards the end of the traveled path observed for samples 2Z and 8. At the beginning of the experiment, the lowest coefficient of friction was observed for the Type 1X samples, which remained low at the end along with the coefficient of friction for the Type 3 and 3X samples. Graphically characterizing the friction forces, regression mathematical models were also developed for the given dependencies. These models indicate a negligible mathematical relationship between the compared variables. From a mathematical perspective, it is not meaningful to investigate the variables compared and create mathematical models, as the polynomial functions of the sixth degree used in the mathematical description resulted in low regression and complex mathematical relationships.

### 3.3. Wear Images of Specimen Surfaces at the Abrasive Inlet (Left) to the Contact Zone and the Abrasive Outlet (Right) from the Containment Zone

**Figure 18:**
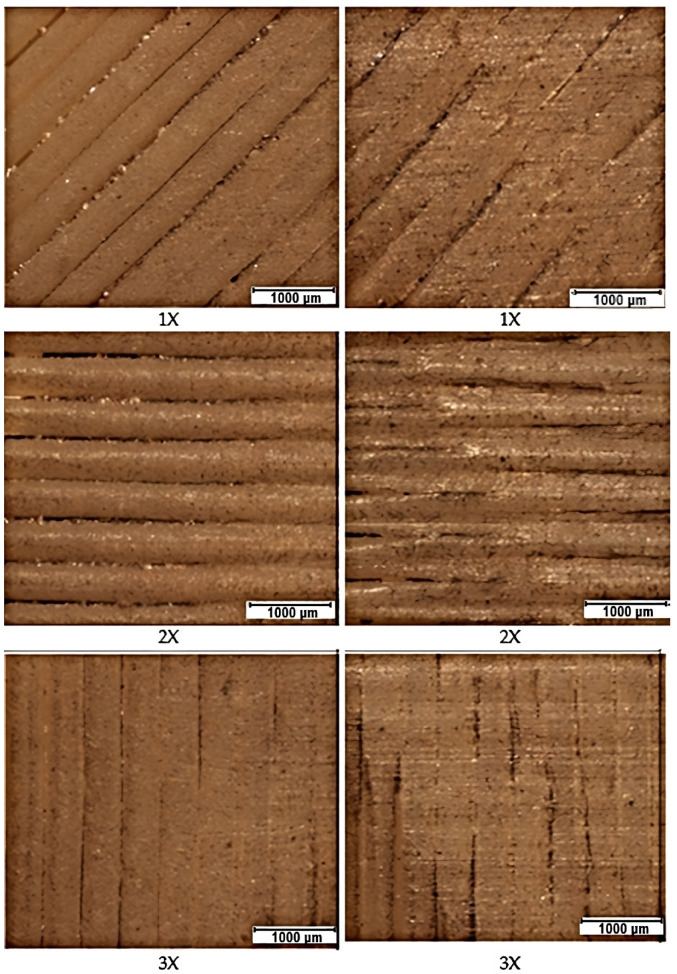
Microscopic images of sample surfaces with X orientation.

**Figure 19:**
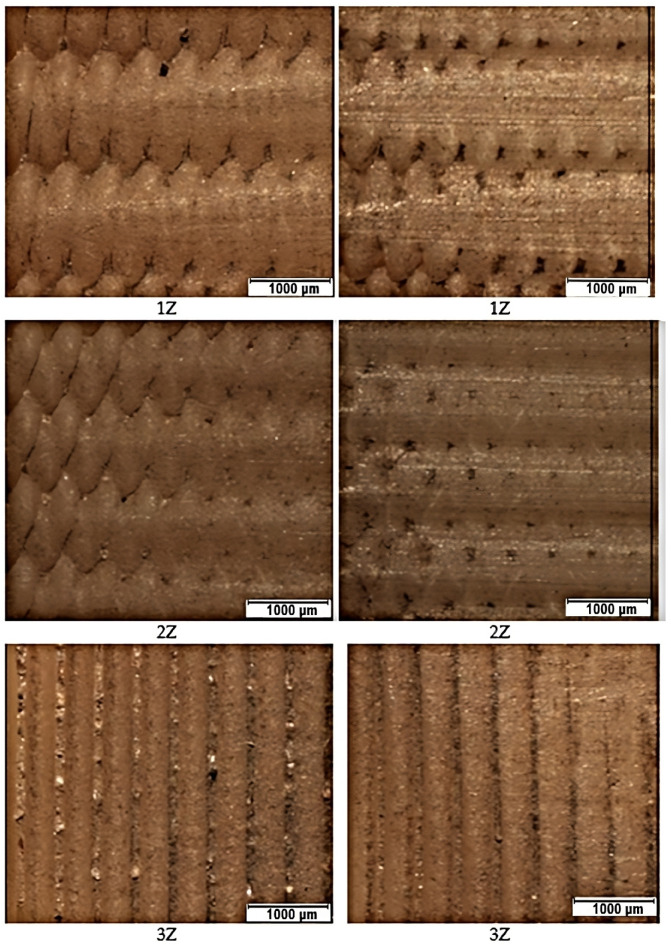
Microscopic images of sample surfaces with Z orientation.

In the Figure 18 and Figure 19 shown below, we have processed the test results from a visual perspective. In the first column, the surfaces before the abrasion test are photographed. The second column contains photos of the surfaces after the input abrasion test (from left to right). The last third column contains the surface of the material at the outlet of the abrasion (from right to left). In these photos, we can see how the abrasive penetrated the different layers of the prints during the test. This can be seen most clearly in specimens with alternating filament deposition. Although the samples were cleaned with the ultrasonic cleaner after testing, we observed residues of abrasive on the surface, especially in orientation Z but also in X orientation.

## 4. Discussion

**Figure 20:**
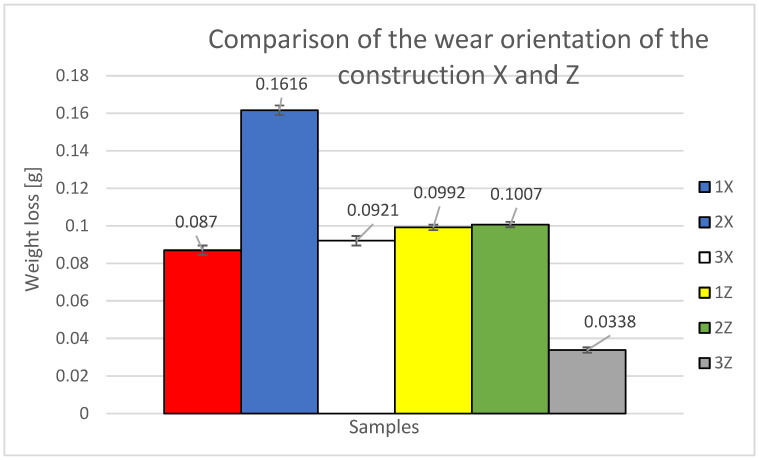
Graphical representation of the comparison of X and Z orientations and standard deviations.

To assess the wear and mass loss of the material, the printing methods of X and Z specimens are compared and evaluated against each other (Table 5 and Figure 20). The specimens were built in layers with a 0.25 mm offset. Additionally, the results of the current study are compared with those of a previous study, where the specimens were built with alternating layers perpendicularly stacked on top of each other. Table 5 presents the average initial weight values of the specimens before the test, the average weight loss in grams, the cumulative loss for each orientation, and the weight loss percentage.

The primary factors investigated were the weight loss in relation to the traveled path and the coefficient of friction. According to Table 5 and Figure 19, it is evident that the highest material loss was observed for the second specimen from orientation X (labeled as 2X), which was constructed at a 90° angle to the tested specimen wall. This indicates that this orientation is the least resistant to abrasive wear, with a material loss percentage of 1.199% of the total specimen weight. The second lowest material loss was recorded for specimens 2Z and 1Z, with orientations of 45° (2Z) and 90° (1Z) relative to the tested specimen wall. These specimens exhibited weight loss percentages of 0.753% and 0.734% of their respective total weights. The third lowest material loss was observed for samples 3X and 1X, with orientations of 45° (3X) and 0° (1X) relative to the tested specimen wall. The weight loss percentages for these samples were 0.668% and 0.624% of their total weights, respectively. The lowest material loss was recorded for the third specimen from the Z orientation (labeled as 3Z), which was constructed at a 0° angle to the tested specimen wall. This indicates that this orientation is the most resistant to abrasive wear, with a material loss percentage of 0.257% of the total specimen weight.

Comparison of weight loss between samples 1X and 1Z revealed a lower weight loss for sample 1X. The experimental results allowed the evaluation of weight loss and friction coefficient as a function of distance traveled.

The comparison reveals that the most abrasion-resistant specimen appears to be the specimen designated 3Z, which has a material loss value of 0.0338 g. When considering the percentage of weight loss relative to the average weight loss in the Z orientation, it corresponds to 14.46% of the average Z orientation loss and 9.921% of the X orientation loss.

The second sample with relatively low weight loss is sample 1X, with a material loss value of 0.087 g. In terms of the percentage of weight loss relative to the average weight loss in the Z orientation, it represents 37.23% of the average Z orientation loss and 25.54% of the X orientation loss.

Next, we have the third sample with relatively low weight loss, which is sample 3X with a material loss value of 0.0921 g. In terms of the percentage of weight loss relative to the average weight loss in the Z orientation, it corresponds to 39.41% of the average Z orientation loss and 27.03% of the X orientation loss.

The following is the fourth sample with increasing weight loss, which is sample 1Z with a material loss value of 0.0992 g. When considering the percentage of weight loss relative to the average weight loss in the Z orientation, it represents 42.45% of the average loss in the Z orientation and 29.12% of the X orientation loss.

The sample labeled 2Z has a relatively low weight loss compared to sample 1Z, with a difference of 19% and a material loss value of 0.1007 g. In terms of the percentage of weight loss relative to the average weight loss in the Z orientation, it corresponds to 43.09% of the average Z orientation loss and 29.56% of the X orientation loss.

The last specimen with the highest percentage of weight loss due to abrasive wear is the specimen labeled 2X, with a material loss value of 0.1616 g. When considering the percentage of weight loss relative to the average weight loss in the Z orientation, it represents 69.15% of the average loss in the Z orientation and 47.43% of the X orientation loss.

The evaluation of the specimen construction system, with alternate layer displacement of 0.25 mm and without alternate layer displacement, reveals the influence of layer orientation on material wear characteristics and the rate of wear by abrasive particles, allowing for predictions regarding their performance in terms of surface and subsurface layer abrasion. Based on this evaluation, specimens with the Z orientation experience less wear and weight loss compared to the X orientation. The Z orientation exhibits a 45.785% lower weight loss compared to the X orientation.

The highest weight loss was observed in sample 3 of the X-oriented samples, where the print paths in both layers were parallel (0°) to the tested sample’s wall. Samples 1 and 2, with deposition paths forming a 45° angle, exhibited weight losses 36.79–45.6% lower than sample 3. Sample 4, with the same deposition strategy for all layers (90°), showed a weight loss reduction of 52.16% compared to sample 3. Sample 5, with alternating layers of perpendicular paths and paths parallel to the test specimen’s wall (90° and 0°), had the lowest weight loss compared to sample 3, up to 52.91% lower.

Based on the results of the weight loss, it can be concluded that using the same strategy in both layers (sample 2, 45°) or a strategy parallel to the direction of wear in both layers (sample 3) led to higher weight loss. On the other hand, employing a strategy in the straight direction for both layers (sample 4, 0°) or a combined strategy resulted in decreased weight loss in the layers (sample 5, 0° and 90°).

Compared with the construction system in the previous study, where the Z orientation had a higher average weight loss of 0.5315 g, indicating a 26.91% increase in wear compared to the X orientation with an average wear of 0.4188 g, significant differences were observed in the behavior of layers deposited using different methods and construction systems towards abrasive wear. Perpendicular layers exhibited higher wear resistance in the X orientation, with a 26.91% increase in wear compared to the Z orientation. In contrast, layers deposited with a 0.25 mm offset in the alternating layers showed the opposite behavior, with X-oriented layers showing a lower abrasive wear resistance.

Based on this evaluation, recommendations are made for 3D printing parts that will experience contact with other surfaces. Specimens with offset alternate layers and X orientation have lower abrasion resistance compared to specimens with a perpendicular and self-aligned construction system in the interlayers. Conversely, specimens built in the Z orientation with a 0.25 mm interlayer offset appear to be more resistant to abrasion.

## 5. Conclusions

The first objective of this work was to investigate and demonstrate the impact of the FFF path generation strategy with a 0.25 mm offset in the alternate layers on the abrasion resistance of the material. It also aimed to compare the wear or material loss among different specimens and between the specimen construction methods in the X and Z orientations. The second objective was to compare the results obtained from the abrasion testing of specimens exposed to a 0.25 mm displacement of alternate layers with the specimens from a previous experiment where the layers were not displaced during construction, i.e., they were stacked perpendicularly on top of each other.

While numerous studies focus on testing the mechanical properties of plastic components, there is a lack of research examining parts printed using FFF technology. This study aims to fill this gap by investigating how the chosen construction orientation and path generation strategy affect the abrasion resistance of 3D printed PEI parts. The objective is to analyze the influence of the construction orientation and coating strategy in the FFF additive manufacturing technique on the evolution of the friction coefficient and wear rate of ULTEM9085™ thermoplastic samples. This will be assessed by measuring the weight loss of the material according to the ASTM G65-16 standard. The PEI material offers the potential to use printed products in demanding applications, making it important to conduct experimental measurements of its abrasive wear resistance.

A total of 18 PEI plastic samples were made by the FFF (FDM) method. The samples were fabricated with two orientations of sample construction and different methods of print path generation. From each path generation method, three samples were made from which average mass loss values were obtained and subsequently worked with in our study.

Based on the data collected and analyzed, a recommendation can be made for parts fabricated using FFF technology with a 0.25 mm offset in alternate layers, particularly when they will be operating in environments where contact with other surfaces or surface abrasion may occur. In such cases, it is advised to construct the parts using the Z orientation construction method. Specifically, the recommended orientation is similar to that used for the 3Z specimen, where the specimen is built in a portrait orientation, and the print paths are oriented at a 0° angle to the specimen wall under test or the location prone to abrasion.

A comparison between specimens with a 0.25 mm offset in alternate layers and specimens constructed with perpendicular alternate layers revealed differences in the durability of the construction systems used. When comparing the X orientation constructions, it was observed that the construction with a 0.25 mm displacement in alternate layers was 22.92% less durable than the system with perpendicular layering. However, in the Z orientation with a 0.25 mm offset in alternate layers, the reverse construction system proved to be up to 127.43% more wear-resistant than the perpendicular layering.

In terms of the total mass loss of material after wear, when considering both orientations and expressed as a percentage, the construction with a 0.25 mm offset in layers exhibited 65.44% greater resistance compared to the construction system with perpendicular layers. These findings can serve as a foundation for future research on the durability of elastomeric and abrasion-resistant products manufactured using FFF technology for component construction. It is anticipated that the abrasion resistance of such materials will present significant challenges in their application [27,28].

## Figures and Tables

**Table 1:** ASTM G65-16 test parameters.

Disc diameter	(mm)	229
Rotations per minute	(RPM)	278
Sample size	(mm)	70 × 20 × 6
Load	(N)	30
Path	(m)	200

**Table 2:** Chemical composition of garnet abrasive [24].

Garnet Fe_3_Al_2_(SiO_4_)_3_	SiO_2_	FeO	Fe_2_O_3_	Al_2_O_3_	CaO	MgO	MnO
	(%)	(%)	(%)	(%)	(%)	(%)	(%)
	41.34	9.72	12.55	20.36	2.97	12.35	0.85

**Table 3:** Slicing and printing parameters.

Parameter	Value
Printing material	ULTEM 9085™
Slicing height	0.254 mm
Width of infill deposition path	0.5 mm
Width of outline deposition path	0.25 mm
No. of outlines in even layer	1
No. of outlines in odd layer	0
Infill deposition path angle	0°, 45°, 90°
Printing head temperature	380 °C
Building chamber temperature	145 °C

**Table 4:** Angle of deposition path alignment to the testing surface.

Sample Type	Strategy	Sample Type	Strategy
1X	45°	1Z	45°
2X	0°	2Z	90°
3X	90°	3Z	0°

**Table 5:** Difference in weight loss within the X and Z sample building direction.

Building Direction	Sample Type	Initial Weight (g)	Weight Loss (g)	Weight Loss (%)
X	1	13.9903	0.0729	0.520
	2	14.0028	0.0847	0.605
	3	13.8603	0.134	0.967
Z	7	14.5401	0.1143	0.786
	8	14.5558	0.1177	0.809
	10	14.5100	0.0988	0.681

## Data Availability

Not applicable.
